# How does the femoral cortex depend on bone shape? A methodology for the joint analysis of surface texture and shape

**DOI:** 10.1016/j.media.2018.01.001

**Published:** 2018-04

**Authors:** A.H. Gee, G.M. Treece, K.E.S. Poole

**Affiliations:** a Department of Engineering, University of Cambridge, Trumpington Street, Cambridge CB2 1PZ, UK; b Department of Medicine, University of Cambridge, Level 5, Addenbrooke’s Hospital, Box 157, Hills Road, Cambridge CB2 2QQ, UK

**Keywords:** Spatial normalization, Textured surfaces, Shape, Statistical parametric mapping

## Abstract

In humans, there is clear evidence of an association between hip fracture risk and femoral neck bone mineral density, and some evidence of an association between fracture risk and the shape of the proximal femur. Here, we investigate whether the femoral cortex plays a role in these associations: do particular morphologies predispose to weaker cortices? To answer this question, we used cortical bone mapping to measure the distribution of cortical mass surface density (CMSD, mg/cm^2^) in a cohort of 125 females. Principal component analysis of the femoral surfaces identified three modes of shape variation accounting for 65% of the population variance. We then used statistical parametric mapping (SPM) to locate regions of the cortex where CMSD depends on shape, allowing for age. Our principal findings were increased CMSD with increased gracility over much of the proximal femur; and decreased CMSD at the superior femoral neck, coupled with increased CMSD at the calcar femorale, with increasing neck-shaft angle.

In obtaining these results, we studied the role of spatial normalization in SPM, identifying systematic misregistration as a major impediment to the joint analysis of CMSD and shape. Through a series of experiments on synthetic data, we evaluated a number of registration methods for spatial normalization, concluding that only those predicated on an explicit set of homologous landmarks are suitable for this kind of analysis. The emergent methodology amounts to an extension of Geometric Morphometric Image Analysis to the domain of textured surfaces, alongside a protocol for labelling homologous landmarks in clinical CT scans of the human proximal femur.

## Highlights

• We consider cohorts of surfaces with scalar data at each vertex: textured surfaces.
• The joint analysis of shape and texture is of interest but also inherently ambiguous.
• The ambiguity may be resolved using homologies to guide vertex correspondences.
• This is an extention of Geometric Morphometric Image Analysis to textured surfaces.
• The method reveals how cortical bone depends on shape in the human proximal femur.

## Graphical abstract

**Figure:**
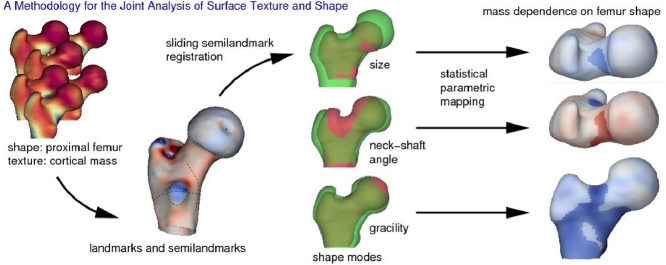

## 1 Introduction

Hip fractures are the most common cause of acute orthopaedic hospital admission in older people (Parker and Johansen, 2006), with their annual incidence projected to rise worldwide from 1.7 million in 1990 to 6.3 million in 2050 (Sambrook and Cooper, 2006). Bone mineral density is currently the imaging biomarker of choice for assessing an individual’s fracture risk, but although it is specific (Johnell, Kanis, Oden, Johansson, Laet, Delmas, Eisman, Fujiwara, Kroger, Mellstrom, Meunier, 3rd, O’Neill, Pols, Reeve, Silman, Tenenhouse, 2005, Kanis, Burlet, Cooper, Delmas, Reginster, Borgstrom, Rizzoli, 2008) it lacks sensitivity (Kanis, Burlet, Cooper, Delmas, Reginster, Borgstrom, Rizzoli, 2008, Kaptoge, Beck, Reeve, Stone, Hillier, Cauley, Cummings, 2008, Sanders, Nicholson, Watts, Pasco, Henry, Kotowicz, Seeman, 2006), missing the majority who go on to fracture. There is now growing evidence that focal, structural weaknesses may predispose a hip to fracture (Mayhew, Thomas, Clement, Loveridge, Beck, Bonfield, Burgoyne, Reeve, 2005, Poole, Mayhew, Rose, Brown, Bearcroft, Loveridge, Reeve, 2010, de Bakker, Manske, Ebacher, Oxland, Cripton, Guy, 2009), with both trabecular and cortical bone playing a role (Holzer, von Skrbensky, Holzer, Pichl, 2009, Verhulp, van Rietbergen, Huiskes, 2008, Poole, Treece, Mayhew, Vaculik, Dungl, Horák, Štěpán, 2012, Kopperdahl, Aspelund, Hoffmann, Sigurdsson, Siggeirsdottir, Harris, Gudnason, Keaveny, 2014).

Cortical bone mapping (Treece, Gee, Mayhew, Poole, 2010, Treece, Poole, Gee, 2012, Treece, Gee, 2015) is an emerging technique for the quantitative analysis of the cortex using clinical CT data. It measures key properties of the cortex, for instance its thickness and mineral density, with high accuracy at several thousand locations across the proximal femur. Each femur is therefore represented as a *textured* surface, with the scalar texture representing the cortical property of interest. Statistical parametric mapping (SPM) (Friston et al., 1994) can then be used to analyse large cohorts of the textured surfaces (Tucholka, Fritsch, Poline, Thirion, 2012, Worsley, Taylor, Carbonell, Chung, Duerden, Bernhardt, Lyttelton, Boucher, Evans, 2009), in order to deduce, for example, how the cortical property depends on age, sex or group. Analyses of this nature have shed light on focal defects that appear to play a role in fracture risk (Treece, Gee, Tonkin, Ewing, Cawthon, Black, Poole, 2015, Poole, Skingle, Gee, Turmezei, Johannesdottir, Blesic, Rose, Vindlacheruvu, Donell, Vaculik, Dungl, Horak, Stepan, Reeve, Treece, 2017, Poole, Treece, Mayhew, Vaculik, Dungl, Horák, Štěpán, 2012), and the efficacy of exercise (Allison et al., 2015) and pharmaceuticals (Whitmarsh, Treece, Gee, Poole, 2016, Poole, Treece, Gee, Brown, McClung, Wang, Libanati, 2015, Whitmarsh, Treece, Gee, Poole, 2015, Poole, Treece, Ridgway, Mayhew, Borggrefe, Gee, 2011) in targeting these defects.

An important step in the SPM pipeline is to *spatially normalize* the textured surfaces, a process which involves registering each surface to a standardized template. Only once the textures have been expressed on a common mesh, is it possible to fit a general linear model and explain the texture at each vertex in terms of the various regressors. In essence, surface registration involves establishing correspondences between the template’s vertices and the vertices of each individual mesh. Inevitably, these correspondences are ambiguous in the barren areas between distinguished features. Different registration algorithms resolve the ambiguity in different ways, in a manner that depends on the surface’s shape. Consequently, SPM analysis of the relationship between a surface’s texture and its shape is problematic, since shape-dependent misregistration induces shape-dependent texture variation which is seen as statistically significant (Gee and Treece, 2014).

**Fig. 1:**
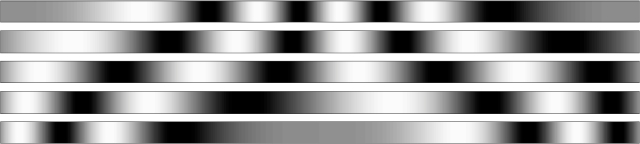
In this one-dimensional example of a textured ribbon, the figure shows five individuals from a population of 201. The population variance can be explained in its entirety by a single linear shape mode (a squash or expansion around the centre, with the ends fixed) and no variance in the texture. An alternative, though less parsimonious, explanation is that there is no variance in the shape but a complex variance in the texture, requiring three linear texture modes to explain 99% of the variance.

To better understand this phenomenon, consider the contrived example in Fig. 1, which shows some one-dimensional textured surfaces. The surfaces are free to deform in the one dimension, so they are best thought of as elastic ribbons. There is no unique way to explain the evident inter-subject variance. At one extreme, we could say that all the ribbons have precisely the same shape, with no elastic stretching or compression, meaning that all the variance is in the texture. At the other extreme, we could say that all the ribbons have precisely the same texture, meaning that all the variance is in the shape. In between these two extremes are a continuum of explanations which involve some shape variation, and also some texture variation that depends on shape. Given this ambiguity, how could we possibly address questions such as “How does the surface’s texture depend on its shape?” And yet such questions are theoretically intriguing and also practically enticing, since femoral shape appears to affect fracture risk (Gregory and Aspden, 2008) and also bone mineral density (Machado et al., 2014). At least in males, the connection between shape and fracture risk is not independent of femoral neck bone mineral density (Ripamonti et al., 2014), hinting at a spatially dependent relationship between gross bone shape and the thickness and density of the cortex.

Returning to the two extreme interpretations of Fig. 1, the shape-only option leads to a compact model that can explain the population variance with a single, linear shape mode: a squash or expansion around the centre, with the ends fixed. This is how the data was generated. In contrast, principal component analysis reveals that the texture-only option requires three texture modes to account for 99% of the population variance. Information parsimony (Davies et al., 2002) is one way to resolve the ambiguity, another being enforced correspondence between distinguished landmarks (Bookstein, 1991). Either way, we need to be clear that any subsequent statistical analysis is entirely predicated on the assumptions used to establish correspondences.

In this paper, we explore these issues in the context of the cortical bone mapping pipeline. Our motivation is to understand how the cortex of the human proximal femur depends on its shape. In Section 2, we review the cortical bone mapping pipeline and describe several different registration algorithms that can be used to spatially normalize the textured surfaces. We design a synthetic data set which sheds light on the systematic misregistration introduced by the various algorithms, and introduce the real human data which we hope to analyse. In Section 3, we perform and discuss a series of experiments on the synthetic data, leading to a novel framework for controlling the correspondence ambiguity. We apply this framework to the real data, producing detailed maps showing the variation of cortical mass with shape across the human proximal femur. After discussing the biomechanical implications of our findings, we draw some conclusions in Section 4.

## 2 Methods

**Fig. 2:**
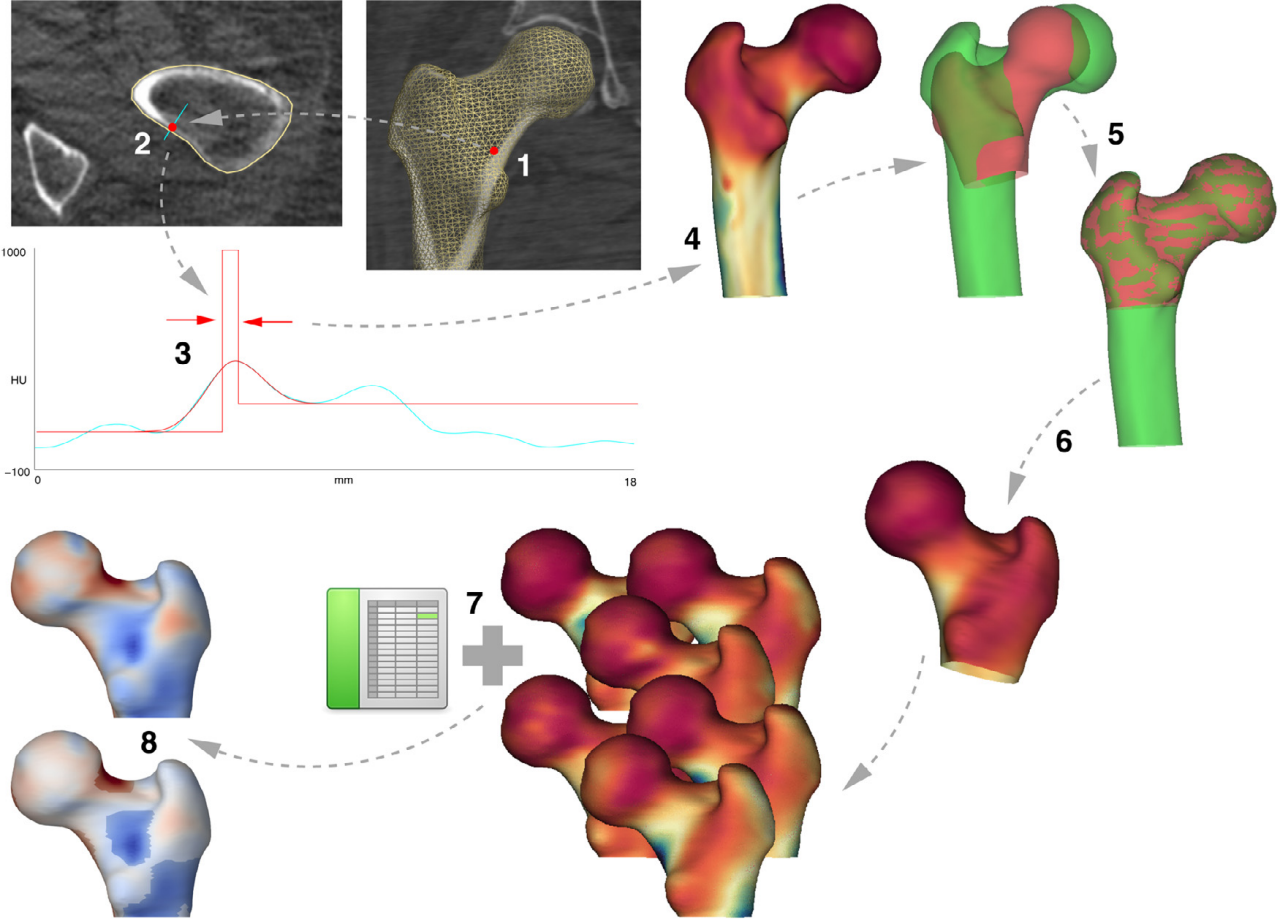
Cortical bone mapping (1–4), spatial registration (5–6) and statistical parametric mapping (7–8). (For interpretation of the references to colour in the text, the reader is referred to the web version of this article.)

The context for this work is a pipeline of processes that enables the characterization and statistical analysis of cortical bone from clinical CT images. Although the pipeline can be applied to any bone with cortical and trabecular compartments, in this work we focus exclusively on the human proximal femur. An overview of the pipeline is presented in Fig. 2. Each stage is described in more detail in the following sections.

### 2.1 Cortical bone mapping

Cortical bone mapping (Treece, Gee, Mayhew, Poole, 2010, Treece, Poole, Gee, 2012, Treece, Gee, 2015) is a technique that estimates the cortical thickness (CTh, cm), cortical bone mineral density (CBMD, mg/cm^3^) and cortical mass surface density (CMSD = CTh  ×  CBMD, mg/cm^2^) at thousands of locations distributed over the proximal femoral surface. The most accurate and precise estimates are for CMSD (Treece and Gee, 2015), which is one of the reasons why we focus on this property in the present work. The other reason is that it is likely to play a significant role in local fracture resistance, accounting as it does for both the amount of cortex (CTh) and the mineralization of said cortex (CBMD).

The starting point for cortical bone mapping is an approximate segmentation of the proximal femur, represented by a triangular mesh with  ∼ 10^4^ vertices (Fig. 2, step 1). At each vertex, the CT data is sampled along a line passing perpendicularly through the cortex (step 2). A model (step 3, red straight lines), that accounts for the imaging blur, is fitted to the data (step 3, cyan curve) so as to minimize the differences between the blurred model (step 3, red curve) and the data. This is repeated at all vertices. The resulting distributions of CTh, CBMD and CMSD can be visualised as texture maps on the femoral surface (in step 4, red is low CMSD while blue is high CMSD). Software to perform the initial segmentation and cortical bone mapping is available for free download.^1^

### 2.2 Spatial registration and the parameterization of shape

For a cohort of size *n*, cortical bone mapping results in *n* texture distributions like the one in Fig. 2, step 4, each expressed on a different triangular mesh (since each individual femur has a different shape and size). Before we can compare these distributions and test how they depend on various regressors, we must first express each distribution on a common mesh. To this end, a canonical femur with 5580 vertices (step 5, red) is rotated, translated and nonrigidly deformed until it aligns with each individual femur (step 5, green). The choice of the surface registration algorithm, and the implications for the subsequent statistical analysis, are the main focus of this paper. Once aligned, the surface texture is mapped from the individual to the canonical femur and smoothed (step 6). The canonical surface mesh (which was constructed by averaging the shapes of several hundred individuals), and software to perform the registration, mapping and smoothing, are available for free download.^2^

Following registration, the *n* sets of deformed canonical vertex coordinates are standardized for location, orientation and scale using Procrustes analysis (Goodall, 1991). This involves translating each specimen to a common origin, scaling to unit centroid size, and then rotating to minimize the sum of the squared distances between the vertices of each specimen and the undeformed canonical mesh. We then rescale each specimen’s vertex coordinates by its centroid size,^3^ and use principal component analysis to build a point-based, statistical shape model from the resulting *n* sets of coordinates. Let **X**_*i*_ be the 16,740-element vector formed by concatenating the coordinates of individual *i*, and let $\hat{X}=\frac{1}{n}\sum_{i=1}^{n}X_{i}$. Then the principal modes of shape variation are the eigenvectors **m**_*i*_ of the sample covariance matrix $\frac{1}{n-1}\sum_{i=1}^{n}\left(X_{i}-\hat{X}\right){\left(X_{i}-\hat{X}\right)}^{T}$. Shape models of this nature are the standard way to obtain compact shape descriptors of individual femurs, which may be represented according to $X_{i}\approx \hat{X}+\sum_{i=1}^{k}S_{i}m_{i}$. For example, setting k=3 produces a 3-element shape vector [*S*_1_ *S*_2_ *S*_3_] accounting for the three most significant modes of shape variation observed in the population. We shall refer to *S_i_* as the *shape coefficients*.

### 2.3 Statistical parametric mapping

Finally, we use SPM, as implemented in the SurfStat package (Worsley et al., 2009), to fit a general linear model (GLM) to the *n* sets of registered texture (Fig. 2, step 7), the aim being to explain the texture at each vertex in terms of regressors of interest (e.g. shape) and also confounding regressors (e.g. age). For example, a GLM explaining the surface texture in terms of the first three shape coefficients would take the form

(1) $$y_{j}=\beta_{0,j}+\sum_{i=1}^{3}\beta_{i,j}S_{i}+\epsilon_{j}$$

where *y_j_* is the surface texture at vertex *j, β*_*i, j*_ are the model coefficients and ϵ_*j*_ is the residual error. For concision, and in common with many statistics packages, we will henceforth refer to GLMs using the more compact model formula, which is $1+\sum_{i=1}^{3}S_{i}$ for the example in Eq. (1). *F* or *t*-statistics can be calculated at each vertex, to test whether the surface texture depends significantly on the regressors, with random field theory furnishing the corresponding *p*-values, corrected for multiple comparisons to control the overall image-wise chance of false positives. The coefficients of the GLM (step 8, top) can be masked to highlight those regions where the effect is statistically significant, for example with *p* < 0.05 (step 8, bottom).

### 2.4 Synthetic data

**Fig. 3:**
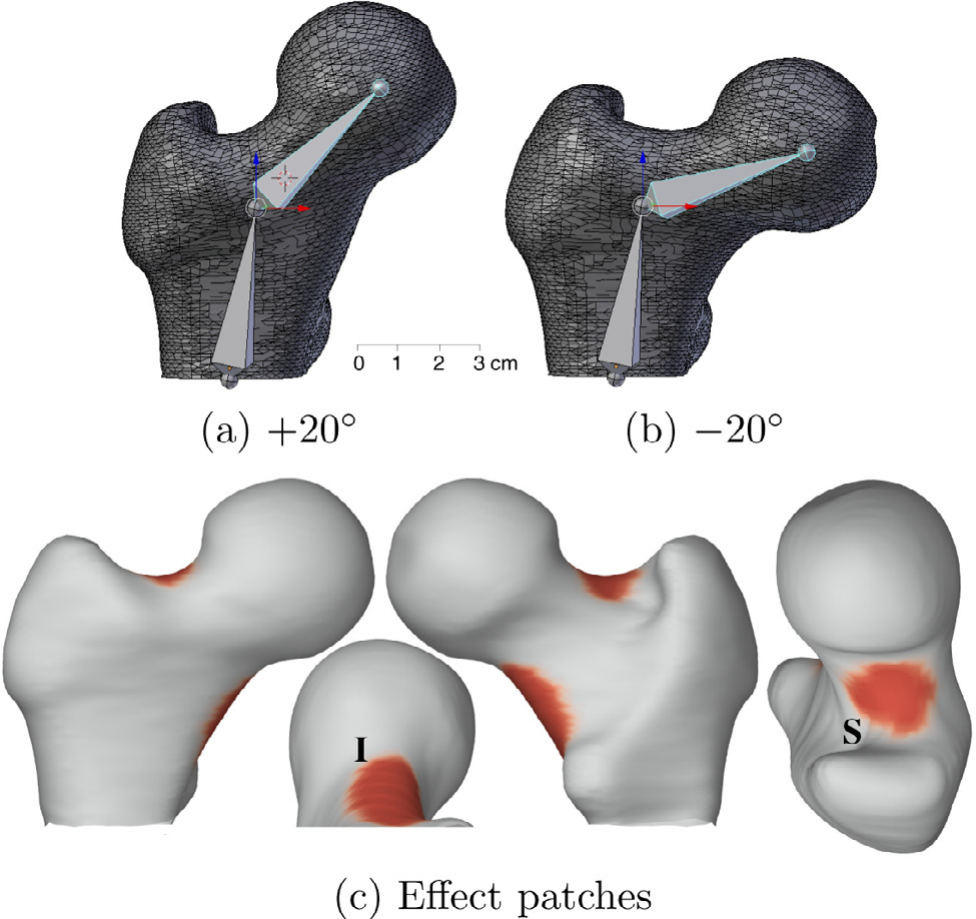
Synthetic femur data. (a) and (b) show the canonical femur mesh at the  ± 20° extremes of bending. Also shown is the internal armature used to define the bending motion. The two “bones” of the armature are hinged where they join, with the mesh “skinned” to the bones. As the armature bends, the mesh vertices are dragged to their new positions by their respective bones. (c) shows the two patches at the superior (S) and inferior (I) femoral neck, where the surface texture was modulated to simulate typical effects. CMSD varies with neck-shaft angle at S, and with gender at I.

To investigate the performance of different approaches to surface registration, we processed synthetic data through the pipeline. The data was generated using the Blender 3D modelling suite (Blender Foundation, Blender Institute BV, Amsterdam, Netherlands). Starting with the canonical femur mesh, we used standard 3D animation techniques to model  ± 20° variations in the neck-shaft angle. Fig. 3(a) and (b) show the internal Blender armature used to achieve this bending motion. We chose to study bending since neck-shaft angle varies significantly in modern human populations (Gilligan et al., 2013), but poses a major challenge to surface registration algorithms. Unless the deformation is constrained by known homologies (Bookstein, 1991), or posed explicitly in terms of known articulations (e.g. Horaud et al., 2009), a registration algorithm will not explain the motion in Fig. 3(a) and (b) as a local deformation at the neck-shaft junction inducing rigid body rotation of the head with respect to the shaft. Most registration objective functions permit affine transformations without penalty, so the motion would instead be modelled as a global shear followed by small, local deformations to bring the surfaces into close alignment.

The synthetic data comprised 41 “male” subjects with neck-shaft angles uniformly distributed in the range ${-20}^{\circ }$ to ${+20}^{\circ }$ at 1° intervals, and 41 “female” subjects with the same shapes as the males. The surface texture of each subject was set initially to the mean CMSD of a real human cohort, as shown in Fig. 11(a). To model fixed effects, the CMSD was reduced by 10% in females and increased by 10% in males at the inferior femoral neck patch in Fig. 3(c). In both the males and the females, the CMSD was reduced by 1% per 1° increase in neck-shaft angle at the superior femoral neck patch in Fig. 3(c): hence, compared with the canonical shape, the ${+20}^{\circ }$ individuals had 20% less CMSD at the superior femoral neck, while the ${-20}^{\circ }$ individuals had 20% more CMSD. Finally, realistic noise was added to each individual’s surface texture, by choosing a set of residuals ϵ_*j*_ (Eq. (1)) at random from a large collection of GLMs fitted to real human data, and adding the residuals to the synthetic surface texture.

### 2.5 Real data

We also studied real human data drawn from two retrospective case-control studies of hip fracture in women. The *Regional Thinning of the Femoral Neck Cortex in Hip Fracture* (FEMCO) study recruited 161 women in the UK, 50 of whom were healthy volunteers attending Addenbrooke’s Hospital, Cambridge. The *Study of Hip Joint in Trauma* recruited 150 women in the Czech Republic, 75 of whom were healthy volunteers attending Homolka Hospital, Prague. The QCT scans were performed on a variety of machines, all including a calibration phantom (five-compartment, Mindways Inc., Austin, TX, USA at Cambridge; two-compartment, Siemens AG, Erlangen, Germany at Prague). Combining the two sets of controls produces a sample size of 125. The FEMCO and Prague data were readily available to the authors, having previously been analysed in fracture case-control studies, and must therefore be viewed as a convenience sample. Demographics for the subjects can be found in Table 1. Informed consent was obtained from all participants.

**Table 1:** Sample size, age, weight and height for the human cohort. The values are given as mean  ±  standard deviation (range).

	n	Age (years)	Weight (kg)	Height (cm)
Females	125	76.8 ± 7.4 (53–98)	66.4 ± 11.1 (40–96)	158.1 ± 6.7 (141–175)

### 2.6 Locally affine registration

**Fig. 4:**
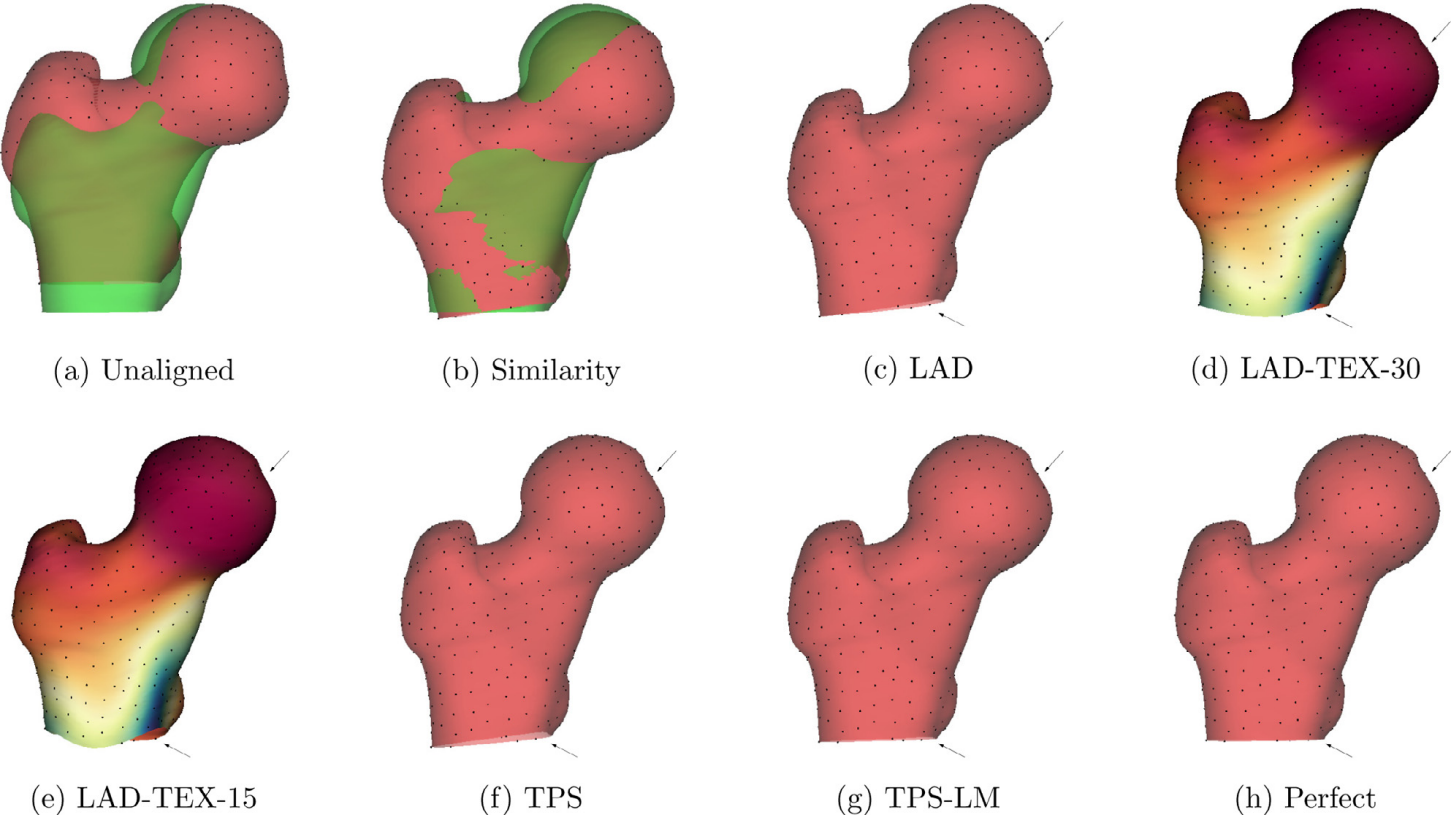
Registrations of the canonical femur (red) to the synthetic ${+20}^{\circ }$ specimen (green). The dots are the surface sliding semilandmarks used in the TPS and TPS-LM algorithms: they also serve to reveal tangential deformation of the surface. For clarity, the target surface (green) is not shown in (c)–(h), since it is almost coincident with the registered canonical surface (red). The arrows are drawn at identical locations in (c)–(h), highlighting obvious registration discrepancies. Close examination of the dots in (c)–(h) reveals significant variation in the tangential alignment computed by the various registration algorithms. The perfect registration in (h) was obtained using the LAD algorithm, but with correct one-to-one vertex correspondences instead of proximity-based correspondences. (For interpretation of the references to colour in this figure legend, the reader is referred to the web version of this article.)

The locally affine registration algorithm of Feldmar and Ayache (1996) finds a nonrigid transformation of surface $M_{1}$ to bring it into alignment with surface $M_{2}$. Associated with each vertex *k* of $M_{1}$ is a set of neighbouring vertices *N_k_*, where each member of *N_k_* lies withing a distance *d* of vertex *k*. The starting point for the algorithm is an approximate alignment computed using the iterative closest point approach of Besl and McKay (1992). This approximate alignment is parameterized by a single, global transformation matrix: we use a similarity transform, comprising rotation, translation and isotropic scaling, as shown in Fig. 4(b). There follows an iterative process to compute the additional, local displacement of each vertex *k* on $M_{1}$. At iteration *i*, every vertex on $M_{1}$ is paired with its closest neighbour on $M_{2}$. Then, for each vertex *k* on $M_{1},$ the rigid transformation ***R***_*k, i*_ is found that minimizes the summed squared distances between the transformed vertices in *N_k_* and their partners on $M_{2}$. The local displacement of vertex *k* is then set to a proximity-weighted average of all the rigid transformations ***R***_*k, i*_ within *N_k_*. At iteration i+1, the closest neighbours and consequent rigid transformations $R_{k,i+1}$ are recomputed, and so on, until convergence. *d* is the algorithm’s only parameter, its effect being to regularise the amount of allowable deformation. Smaller values of *d* permit more deformation and closer alignment of the two surfaces, while larger values of *d* favour smooth displacement fields over alignment accuracy. We shall refer to this algorithm using the acronym LAD (locally affine deformation). We set the parameter *d* to 15 mm for all the shape-driven LAD experiments in this paper.

LAD is an uncomplicated example of a class of shape-driven registration algorithms that apply local, nonrigid transformations on top of a global rigid or affine alignment. More sophisticated exemplars have been developed within a robust mathematical framework to guarantee diffeomorphic deformations (Joshi, Miller, 2000, Beg, Miller, Trouvé, Younes, 2005). A common limitation of all such approaches is that they are sensitive to the initial, global alignment (Ashburner, 2007). This is evident in Fig. 4(c), where there is obvious tangential misalignment at the truncated femoral shaft and also at the fovea capitis (compare the arrows, drawn in identical positions, in Fig. 4(c) and (h)), caused by incorrect proximity-driven vertex correspondences that have their origins in Fig. 4(b).

Nevertheless, the simple LAD algorithm serves our illustrative purposes well. For *d* ≥  15 mm, we never observed any surface folding. Moreover, it is straightforward to modify the LAD algorithm to incorporate an element of texture-driven registration. After initial LAD convergence, further iterations may be performed with vertices on $M_{1}$ paired not with their nearest neighbours on $M_{2},$ but instead with the vertex on $M_{2},$ within a reasonable search range (we used 3mm), that has the most similar texture. This has the effect of modifying the initial LAD registration to reduce the dissimilarity between the aligned texture fields. We refer to this variant using the acronym LAD-TEX-*d*, where *d* is the parameter (in mm) used to define the LAD vertex neighbourhoods. By varying *d*, we can control how much extra deformation to allow: large values of *d* imply a smooth displacement field, whereas small values of *d* allow more deformation. Texture-driven algorithms of this nature have been recommended for the registration of textured surfaces (Sidorov, Richmond, Marshall, 2011, Lin, Lai, Martin, Jin, Cheng, 2016).

Fig. 4(d) and (e) show the canonical mesh, textured with the cohort average CMSD of Fig. 11(a), registered to the synthetic female ${+20}^{\circ }$ specimen using the LAD-TEX algorithm with d= 30 mm and d= 15 mm respectively. Had the two texture fields been identical, the LAD-TEX algorithm would have brought them into perfect alignment. But the textures differ at both of the patches in Fig. 3(c) and also by virtue of the added noise. While the fovea capitis is now better aligned (arrows), attempting to align the different textures at the inferior femoral neck has resulted in obvious registration errors at the truncated shaft.

### 2.7 Registration using sliding semilandmarks

The sliding semilandmark algorithm, originally developed for planar morphometry (Bookstein, 1991, Bookstein, 1997) and subsequently extended to surfaces (Gunz et al., 2005), is a mainstay of the geometric morphometrics community. The method requires a set of point landmarks on surface $M_{1}$ and a matching set on surface $M_{2}$. The landmarks fall into three categories: homologous points that are known to correspond on the two surfaces (e.g. the centre of the fovea capitis), points on corresponding curves (e.g. the linea aspera) and points that lie on the surfaces but are otherwise undistinguished. The sliding semilandmark algorithm finds the thin plate spline (TPS) that warps the landmarks on $M_{1}$ so that they align perfectly with their partners on $M_{2}$. In so doing, it reconfigures the landmarks on $M_{2}$ so as to minimize the TPS bending energy. Curve-based landmarks are allowed to slide in one dimension, tangentially to their curves, while surface-based landmarks are allowed to slide in two dimensions, tangentially to the surface. These sliding landmarks are often referred to as semilandmarks. Homologous point landmarks are true landmarks and are not allowed to slide.

While homologous point and curve landmarks generally need to be located by an expert (Gunz and Mitteroecker, 2013), surface-sliding semilandmarks can be distributed automatically, at evenly spaced locations on the meshes, and are sufficient to align the two surfaces. We used a total of 476 such semilandmarks, the dots in Fig. 4. We shall refer to this automatic registration method using the acronym TPS.

**Fig. 5:**
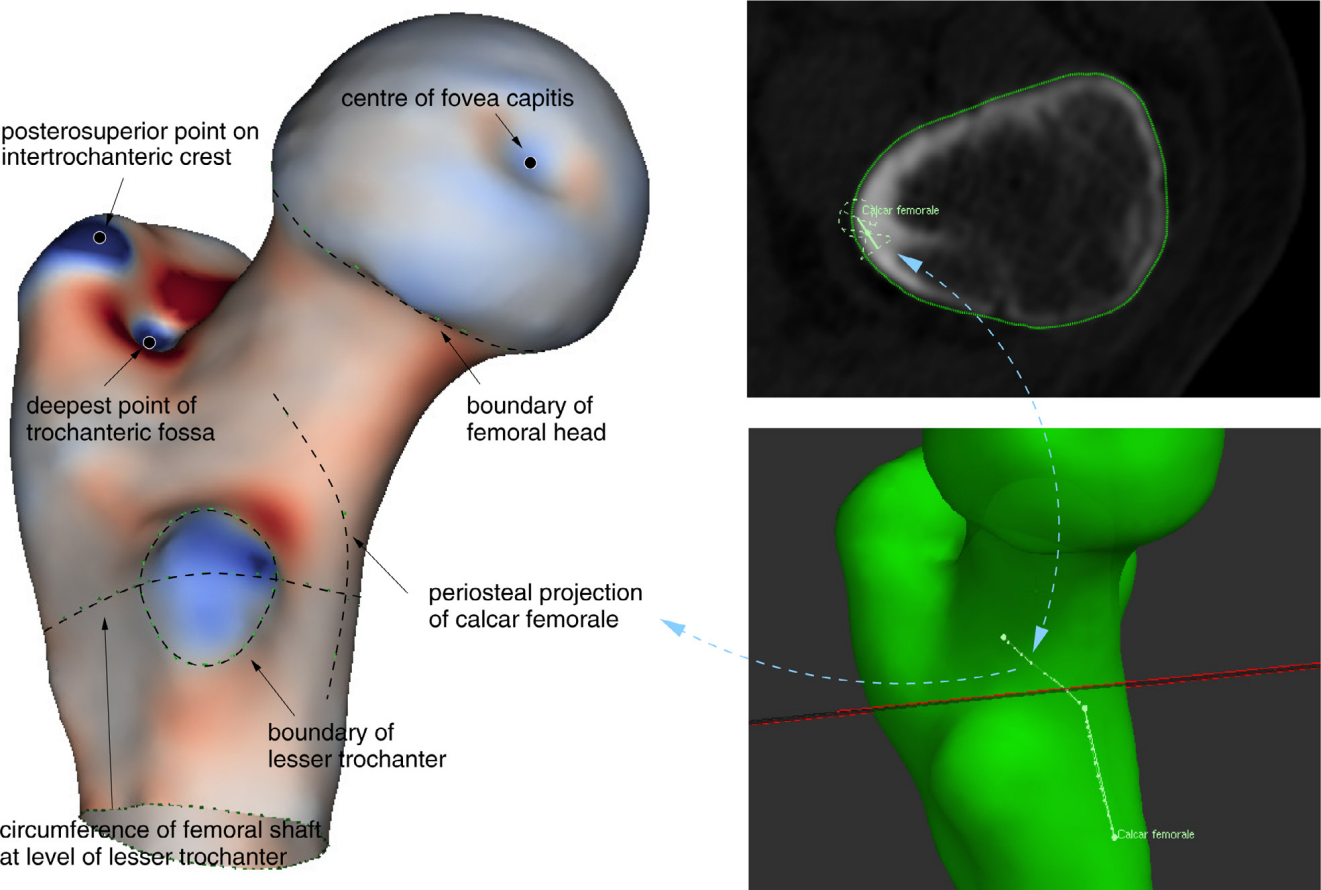
Point and curve landmarks used in the TPS-LM registration algorithm. Most of these landmarks are placed manually or semi-automatically with reference to the Gaussian curvature of the mesh. In the rendering on the left, Gaussian curvature is shown on a red–blue scale, with blue representing positive values and red representing negative values. The calcar femorale are traced manually on the green mesh (bottom right), the resulting curve is projected back into the original CT data (top right), and the curve is then edited on the green mesh until the projections align well with the calcar in the CT data. (For interpretation of the references to colour in this figure legend, the reader is referred to the web version of this article.)

We also placed homologous point and curve landmarks on distinguished features of the proximal femur that are readily identified in low resolution, clinical CT images. Following Harmon (2007), we located point landmarks at the centre of the fovea capitis, the posterosuperior point on the intertrochanteric crest and the deepest point of the trochanteric fossa. These points are especially easy to identify when the surface rendering is shaded with the local Gaussian curvature, as in Fig. 5. We added a curve landmark at the periosteal projection of the calcar femorale, which is readily apparent in the CT data, as shown at the right of Fig. 5. We added further curve landmarks around the boundaries of the femoral head and the lesser trochanter, defined by the intersection of the surface with the best-fit planes that partition areas of positive and negative Gaussian curvature (i.e. blue-to-red transitions in Fig. 5). We added a final curve landmark around the femoral shaft at the level of the lesser trochanter, defined by the intersection of the surface with the plane that passes through the centre of the segmented lesser trochanter and whose normal is parallel to the shaft, the shaft direction being estimated automatically as the best mutual perpendicular to the triangles at the distal end of the mesh.

While none of these landmarks would pass the most stringent test of true biological homology (the so-called Type I landmarks of Bookstein, 1991), they serve our purpose in that their locations are ostensibly unbiased by the *gross* shape of the specimen. For example, neck-shaft angle and femoral neck length do not obviously influence the location of any of these landmarks. They therefore play a potential role in removing systematic misregistration effects that depend on gross shape. We shall refer to their use in the sliding semilandmark algorithm using the acronym TPS-LM.

In Fig. 4(g), the TPS-LM registration is virtually indistinguishable from the ground-truth perfect registration in Fig. 4(h). In contrast, the automatic TPS algorithm in Fig. 4(f) shows evidence of shearing at the truncated femoral shaft. This is unsurprising, since affine transformations incur no TPS bending energy penalty (Gunz et al., 2005), so are preferred to bending in the absence of homologous landmark constraints.

## 3 Experiments, results and discussion

### 3.1 Synthetic data

#### 3.1.1 Shape-texture variance trade-off

**Fig. 6:**
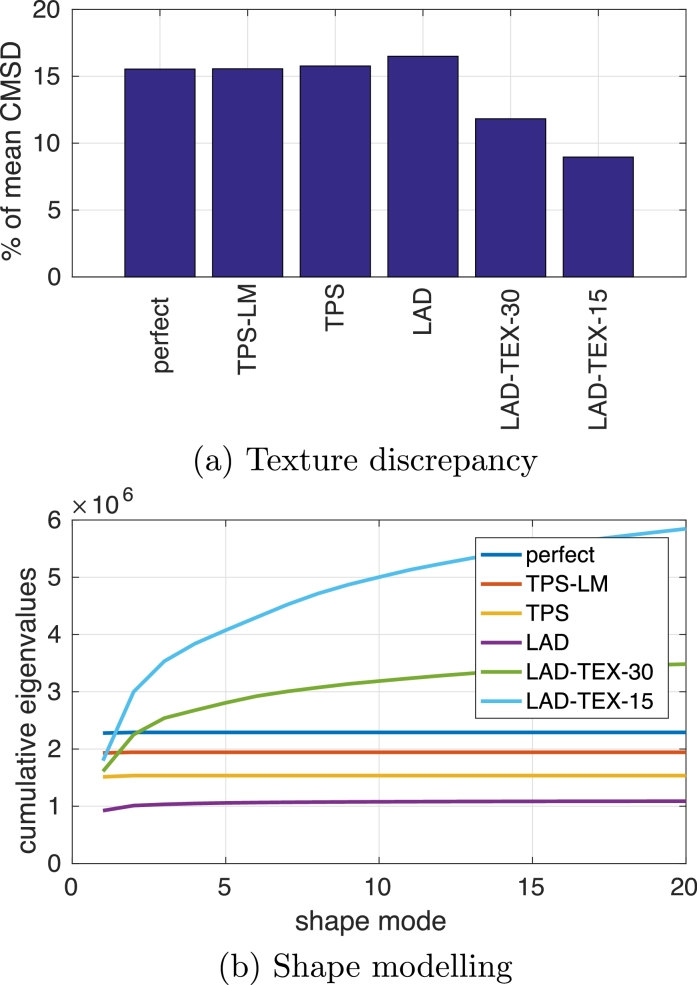
Registration performance indicators for the synthetic data. (a) shows the average, post-registration root mean square texture discrepancy, expressed as a percentage of the mean CMSD. (b) shows the cumulative eigenvalues (reflecting cumulative shape variance) of the statistical shape models for the first 20 shape modes. (For disambiguation of the lines in (b), the reader is referred to the colour web version of this article.)

We registered the canonical femur mesh, textured with the mean CMSD in Fig. 11(a), to the 82 synthetic specimens using the six different registration algorithms introduced in Section 2. Fig. 6(a) shows the post-registration texture misalignment, quantified as the root mean square discrepancy between the canonical CMSD and each individual’s CMSD, and expressed as a percentage of the mean CMSD. When perfectly aligned, this discrepancy is around 15%, since there is noise and also systematic texture variation depending on gender and shape. The LAD-TEX-30 algorithm performed as expected, finding imperfect alignments that nevertheless reduce the mean texture discrepancy to around 12%. The LAD-TEX-15 algorithm was able to introduce further local shape deformation and achieve a mean texture discrepancy of around 9%.

We then built statistical shape models using the six sets of vertex displacements. Fig. 6(b) shows the cumulative eigenvalues, reflecting the amount of shape variance embodied in the first 20 modes of the models. Taken together, Fig. 6(a) and (b) show the trade-off between attributing variance to texture or shape: without exception, as the post-registration texture variance decreases, so the total shape variance increases. Note that the differences in asymptotic shape variance reflect mostly *tangential* surface deformation, since all the registration algorithms bring the surfaces into reasonable normal alignment.

**Fig. 7:**
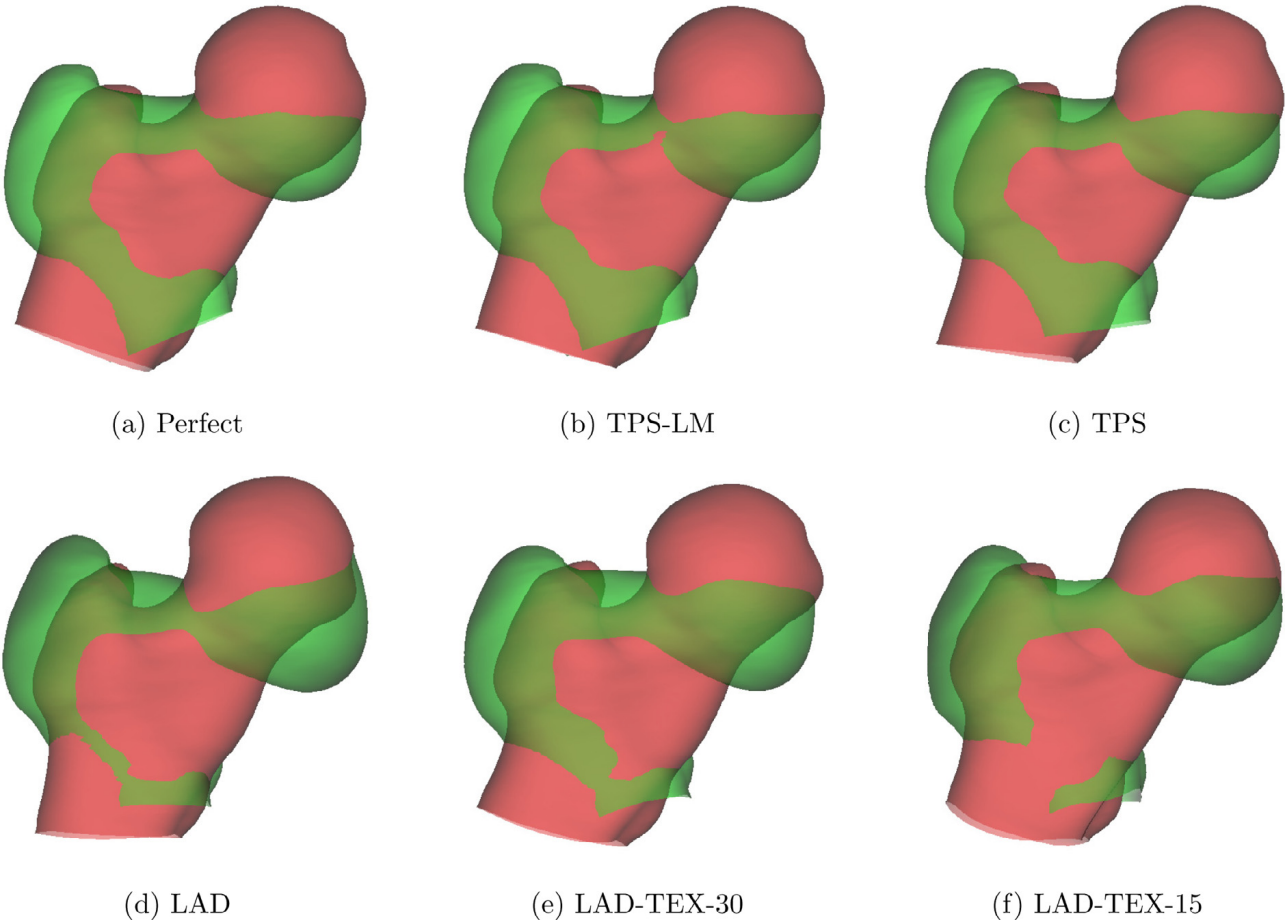
The first mode of the statistical shape models,  ± 3 standard deviations. Red is +3 standard deviations, green is −3 standard deviations. The  ± 3 standard deviation range exceeds the $\pm \sqrt{3}$ standard deviation range of the uniform distribution from which the data was generated, but the extrapolated range better illustrates the differences between the various models. (For interpretation of the references to colour in this figure legend, the reader is referred to the web version of this article.)

Fig. 7 shows the first shape mode for each of the six models. With perfect registration and also with the TPS-LM algorithm, this mode (the only significant mode) captures the ground-truth bending deformation. In contrast, inspection of the truncated shaft reveals how the TPS and LAD algorithms interpret the motion as mostly shearing. The LAD-TEX algorithms introduce more complex deformations that reduce the post-registration texture variance.

#### 3.1.2 Statistical parametric mapping

**Fig. 8:**
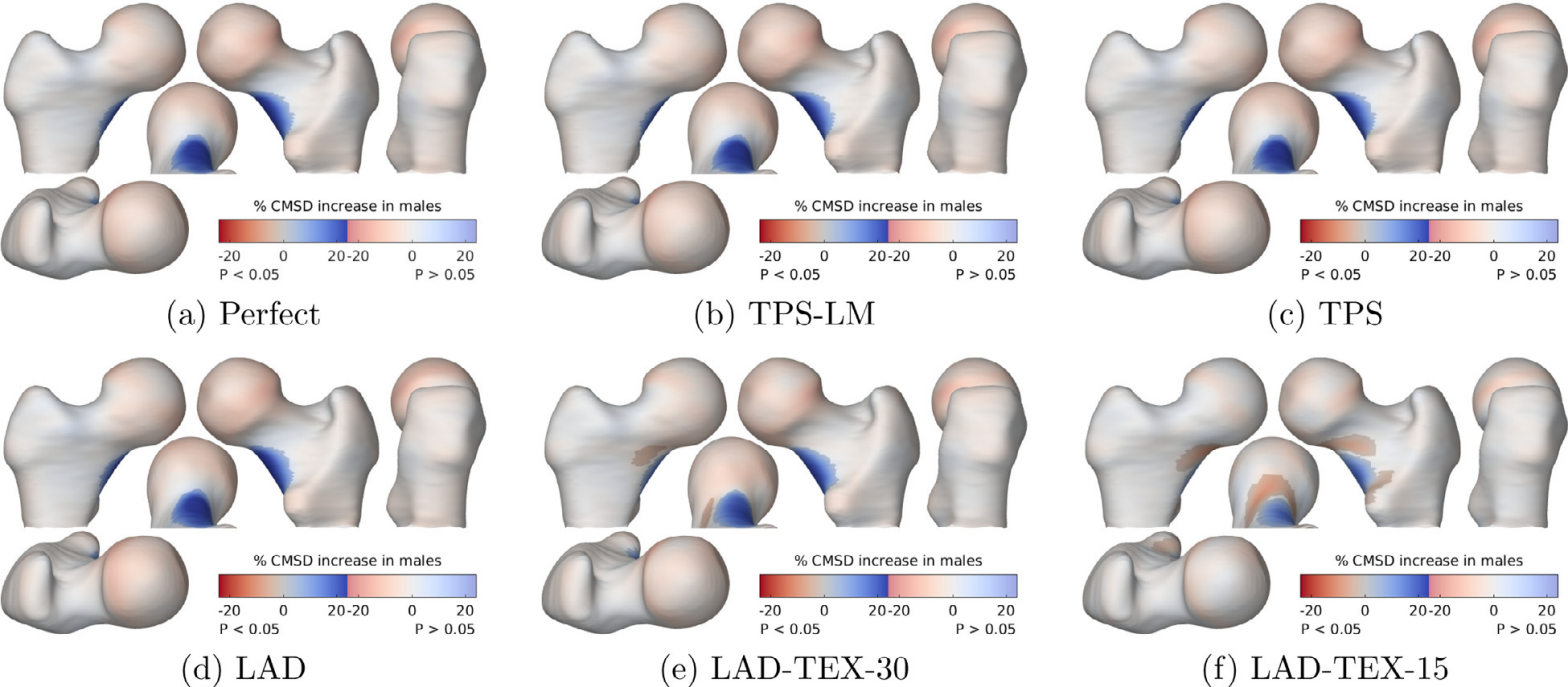
SPM analysis of the relationship between CMSD and gender. The GLM fitted was $1+\text{Gender}+\sum_{i=1}^{3}S_{i}$. The maps show the percentage increase in CMSD for males compared with females, masked to highlight regions where the effect is statistically significant at the 5% level. The significance test was based on the extent of connected clusters exceeding an uncorrected *p*-value threshold of 0.001.

**Fig. 9:**
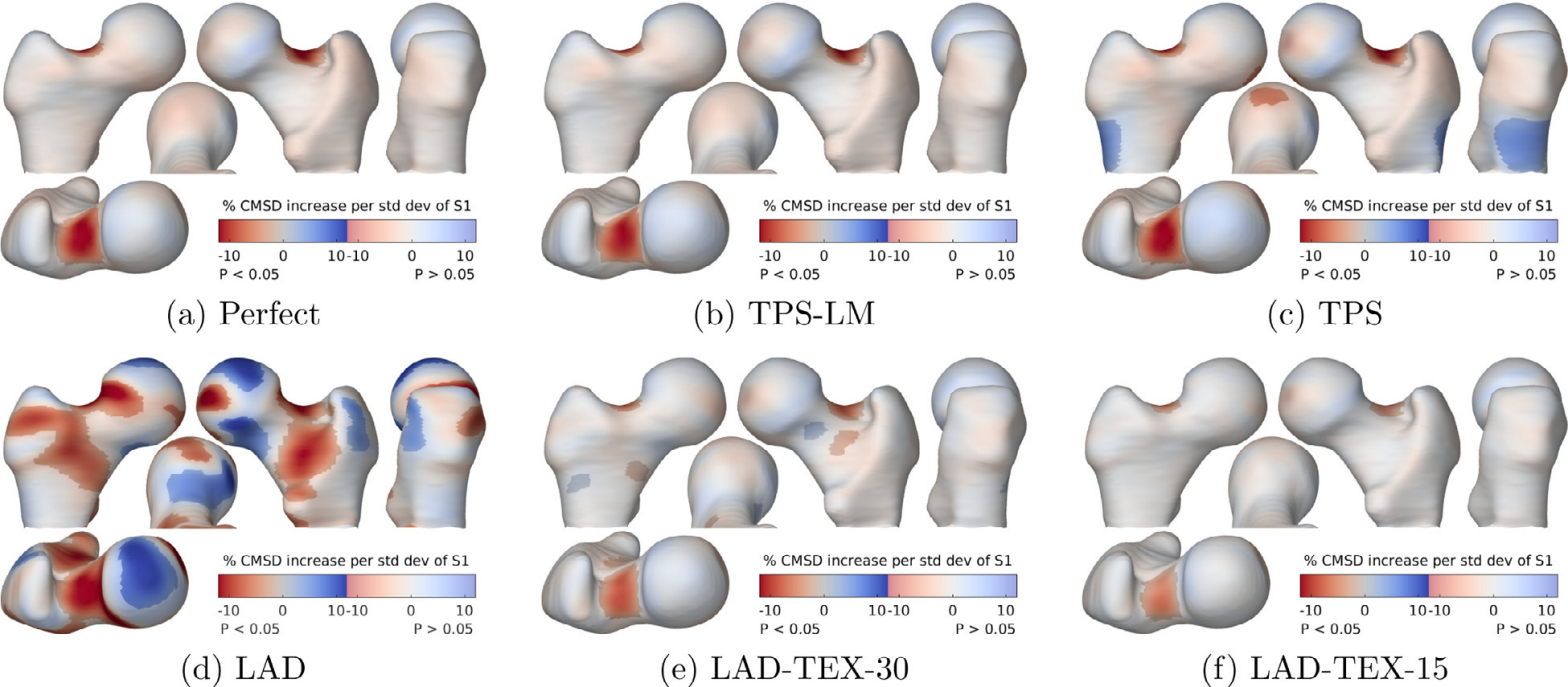
SPM analysis of the relationship between CMSD and shape. The GLM fitted was $1+\text{Gender}+\sum_{i=1}^{3}S_{i}$. The maps show the percentage increase in CMSD per standard deviation increase in *S*_1_, masked to highlight regions where the effect is statistically significant at the 5% level. The significance test was based on the extent of connected clusters exceeding an uncorrected *p*-value threshold of 0.001.

Figs. 8 and 9 show the results of SPM with the GLM $1+\text{Gender}+\sum_{i=1}^{3}S_{i}$. Recall that the ground-truth texture varied by 20% with gender at the inferior femoral neck, and by 1% per 1° variation in neck-shaft angle (which equates to 11.5% per standard deviation) at the superior femoral neck. Since the sample size is arbitrary, the *p*-masks in Figs. 8 and 9 provide nothing more than a correspondingly arbitrary threshold for comparing the performances of the registration algorithms. They do not reflect replicability of the study, as would normally be the case.

The key point to note from Fig. 8 is the success of the TPS, TPS-LM and LAD algorithms in recovering the correct dependence of CMSD on gender, despite the registration ambiguity and the different ways the three algorithms have modelled the shape variation. The reason for this is that these three algorithms are driven entirely by shape; so the way they resolve ambiguity in vertex correspondence depends only on shape; so the ambiguity is manifested only in the dependence of CMSD on shape, and not on gender which is independent of shape. It would appear that statistical analysis of surface texture can be insensitive to the choice of registration algorithm, as long as the regressor is independent of shape (this would need to be demonstrated empirically), the registration algorithm is purely shape-driven, and the GLM includes shape coefficients as confounding variables. This is a key finding of this study.

Quite different results are obtained by the LAD-TEX algorithm, since the vertex correspondences now depend on texture and therefore gender. The results in Fig. 8(e) and (f) conflate the true gender effect with the LAD-TEX algorithm’s efforts to reduce the texture variance by registering the males and females in systematically different ways. Note how this results in an attenuated, bipolar gender effect that extends beyond the ground-truth patch at the inferior femoral neck.

Importantly, with the LAD-TEX algorithm, the difference between males and females is no longer confined to the surface texture, but also embedded in the shape coefficients. Statistical analysis of the surface texture alone, as in Fig. 8(e) and (f), is of questionable value. This phenomenon is well understood and has been much discussed in the literature, particularly in the context of voxel-based morphometry (Ashburner, Friston, 2001, Bookstein, 2001b, Bookstein, 2001a), an SPM variant for analysing anatomical shape. At least one SPM workflow has evolved from using texture-driven registration (Li et al., 2009) to purely shape-driven registration (Carballido-Gamio, Harnish, Saeed, Streeper, Sigurdsson, Amin, Atkinson, Therneau, Siggeirsdottir, Cheng, III, Keyak, Gudnason, Khosla, Harris, Lang, 2013b, Carballido-Gamio, Harnish, Saeed, Streeper, Sigurdsson, Amin, Atkinson, Therneau, Siggeirsdottir, Cheng, III, Keyak, Gudnason, Khosla, Harris, Lang, 2013a).

Unfortunately, there is no simple way of marginalising the correspondence ambiguity when the regressor of interest depends on shape. In Fig. 9, there is no consensus regarding the relationship between CMSD and shape, with only the TPS-LM algorithm agreeing with the ground truth. At the other extreme, the LAD results in Fig. 9(d) exhibit large areas of artefact, caused by tangential misalignment of the texture field, with the misalignment depending systematically on shape (Gee and Treece, 2014). The different registration algorithms have modelled the shape variation in different ways, leading to different answers to the question “How does surface texture depend on shape?”

#### 3.1.3 Parsimonious modelling of shape and texture

This begs the question: is it even feasible to construct joint models of texture and shape, and study their covariance, in an unambiguous and automatic manner? What is it about the ground-truth solutions in Figs. 7(a)–9(a) that objectively signals their correctness compared with the other solutions? The literature points to a response based on Occam’s law of parsimony: we seek the most parsimonious interpretation of the data, and we predicate our statistical analyses (e.g. how does surface texture depend on shape?) on this principle. In this way, shape ambiguity is resolved in an explicit and quantifiable manner: the correct solution is the one that produces the most compact model, a model which we also expect to have good specificity and ability to generalise (Davies et al., 2010).

There have been attempts to build statistical shape models under minimum description length (MDL) optimality criteria, originally considering only plane contours (Davies et al., 2002) but later encompassing also surfaces (Heimann, Wolf, Williams, Meinzer, 2005, Davies, Twining, Cootes, Taylor, 2010). There has been some preliminary work at extending the paradigm to cover appearance as well as shape (Baker, Matthews, Schneider, 2004, Marsland, Twining, Taylor, 2008). Myronenko and Song (2010) describe a way of registering textures so as to minimize the *complexity* of the residual, rather than the residual itself. However, MDL model building is computationally and theoretically challenging. The search space of possible models is vast, reflecting the myriad permutations of correspondences between thousands of mesh vertices across hundreds or thousands of specimens. A further difficulty is to formulate a robust, information-theoretic objective function. The synthetic femur experiment and the 1D example in Fig. 1 demonstrate that this is not just a matter of selecting the model with the fewest shape modes, or the lowest overall shape variation, or the lowest texture variation. Instead, the cost function needs to account for the number of modes and also the information content of the modes, allowing for correlations between neighbouring vertices (Thodberg, 2003). A further consideration is the degree of parsimony achievable by boundary point representations followed by Euclidean principal component analysis, compared with alternatives such as medial descriptors followed by principal geodesic analysis (Fletcher et al., 2004).

Notwithstanding the significant theoretical and practical difficulties, it is by no means clear that meaningful statistical inference can follow from automatic, parsimony-driven modelling. For example, consider the perfect and TPS solutions in Fig. 6, Fig. 7, Fig. 8, Fig. 9. Which interpretation of the data is more parsimonious? Both models involve just one significant shape mode. The shape variance embodied by this mode is less in the TPS solution than in the perfect solution, since the shearing motion in Fig. 7(c) involves, on average, less vertex displacement than the bending motion in Fig. 7(a). We might therefore favour the TPS model. Set against this is the greater complexity of the texture variation in Fig. 9(c) compared with Fig. 9(a): two effect regions versus one. Choosing between these two models based on information parsimony appears to be balanced on a knife-edge, and yet they lead to different and incompatible deductions about the population: either the femurs differ in shape through bending, and the CMSD depends on the degree of bending at the superior femoral neck; or the femurs differ in shape through shearing, and CMSD depends on the degree of shearing at the superior femoral neck and the lateral femoral shaft.

#### 3.1.4 Geometric morphometric image analysis

We would argue that neither of these interpretations can be deemed to be “correct” unless the registration is constrained by known, axiomatic homologies. We therefore lend our voice to the argument of Gunz and Mitteroecker (2013), who say, when discussing automatic, “homology-free” registration algorithms:

... the point homology across specimens, which is “enforced” by the experienced morphometrician measuring semilandmarks on curves and surfaces manually, is no longer guaranteed. As a result, sample averages and variances may be meaningless and biologically not interpretable. If one aims to go beyond the mere discrimination of groups and tries to identify the biological factors underlying shape differences, the time spent digitizing curves and surfaces as semilandmarks is almost always worthwhile.

We conclude, therefore, that only the TPS-LM algorithm has a role to play in analysing the variation of CMSD with shape. The combination of landmark-based registration with statistical shape and appearance modelling has been styled *Geometric Morphometric Image Analysis* (GMIA) and applied to the analysis of planar shapes and images (Mayer, Metscher, Mller, Mitteroecker, 2014, Mayer, Windhager, Schaefer, Mitteroecker, 2017). This paper describes the first extension of GMIA to the domain of textured surfaces. It must be stressed that GMIA is no panacea: manual landmarking requires both expertise and time, and there remains the question of how to interpret the data in the barren regions between landmarks. We shall return to this point in the next section.

### 3.2 Real data

We registered the canonical surface using the TPS-LM algorithm to 173 proximal femurs segmented from the real CT data. CMSD was estimated for each specimen, smoothed with an approximately 10mm full-width-half-maximum filter (in order to ensure compatibility with the Gaussian random field theory that underpins SPM) and then mapped onto the registered canonical femur. Both left and right femurs were available for 48 of the subjects: for these, the mapped data was averaged on the canonical mesh. Taken together with the 77 sets of unilateral data, the end result was 125 sets of mapped data from 125 individuals. We then built statistical shape models using the TPS-LM vertex displacements. Finally, statistical analysis was performed using SPM with the GLM $1+\text{Age}+\sum_{i=1}^{6}S_{i}$.

**Fig. 10:**
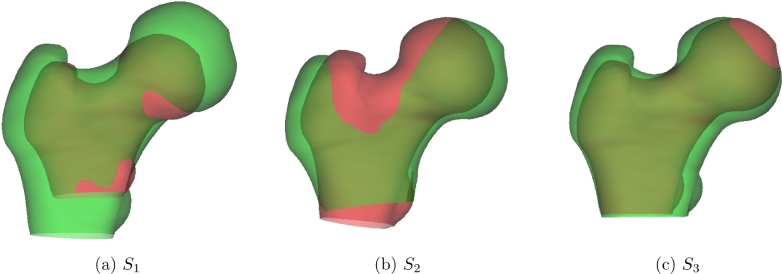
The first three shape modes of TPS-LM registered human data,  ± 3 standard deviations. Red is +3 standard deviations, green is −3 standard deviations. The three modes account for 65% of the shape variation observed in the population. It is apparent that *S*_1_ corresponds roughly to femur size, *S*_2_ to neck-shaft angle and *S*_3_ to gracility. (For interpretation of the references to colour in this figure legend, the reader is referred to the web version of this article.)

**Fig. 11:**
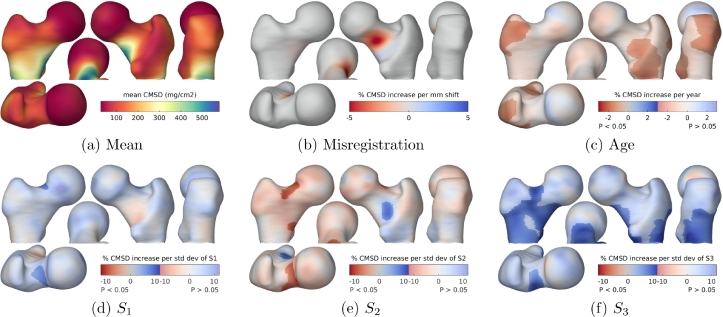
SPM analysis of the human data. The GLM fitted was $1+\text{Age}+\sum_{i=1}^{6}S_{i}$. The maps in (c)–(f) show the percentage increase in CMSD per year of age and per standard deviation increase in $S_{i},i\in \left\{1\ldots 3\right\},$ masked to highlight regions where the effect is statistically significant at the 5% level. The significance test was based on the extent of connected clusters exceeding an uncorrected *p*-value threshold of 0.001. For comparison with (e), the map in (b) shows, for the synthetic data, the percentage increase in CMSD per millimeter misregistration along the calcar femorale. (For interpretation of the references to colour in the text, the reader is referred to the web version of this article.)

Fig. 10 shows the first three modes of the statistical shape model, and Fig. 11(c)–(f) show the regions where CMSD varies significantly with age and shape. While many of these effects have been predicted and observed by other researchers (Machado, Fernandes, Zymbal, Baptista, 2014, Ripamonti, Lisi, Avella, 2014), albeit in terms of bone mineral density at broad regions of interest, the apparent increase in CMSD with increasing neck-shaft angle at the calcar femorale is a new finding and therefore warrants greater scrutiny. Homology in this area is enforced by curve landmarks only: hence, the alignment *along* the trajectory of the calcar femorale is relatively unconstrained and determined in the most part by the minimum bending energy criterion of the TPS-LM algorithm. Might this effect be a systematic misregistration artefact, akin to the arbitrary shape effects demonstrated with the synthetic data in Fig. 9?

We offer two reasons why this is most likely a real, physiological effect and not a misregistration artefact. Firstly, in the synthetic data experiments, the TPS-LM algorithm did not introduce any significant artefacts at the calcar region in response to bending: see Fig. 9(b). A more rigorous argument uses the TPS-LM algorithm to quantify what systematic misregistration along the trajectory of the calcar femorale would look like. In a new set of synthetic experiments, the canonical femur with the mean CMSD texture was registered to a population of identical femurs with identical textures, but with the calcar landmarks changed from curve landmarks to point landmarks. These point landmarks were then systematically displaced along the calcar trajectory by varying amounts, inducing precisely the systematic misregistration we are trying to rule out in Fig. 11(e). Fig. 11(b) shows the results of this experiment: systematic misregistration would cause a bipolar CMSD effect at the calcar region, with its focus adjacent to the femoral head, and not the monopolar effect in Fig. 11(e), with its focus closer to the lesser trochanter. We believe that this sort of detailed analysis is essential before confirming any systematic dependence of surface texture on shape.

Similar considerations lend support to the authenticity of the other effects in Fig. 11(c)–(f). The *S*_2_ and *S*_3_ effects at the superior femoral neck are reasonably well constrained by the homologous point landmark at the trochanteric fossa and the curve landmark at the boundary of the femoral head. Observe how the CMSD gradient is roughly perpendicular to the neck-head boundary, so misregistration tangential to the curve landmark will not induce significant effects at the superior femoral neck. The *S*_3_ effect, covering a large part of the proximal femur, is almost entirely monopolar and so cannot be explained by smooth, systematic misregistration. Although the finer details remain uncertain, particularly in those regions some distance away from the nearest landmark, there is undoubtedly a widespread increase in CMSD with increasing gracility. The age and *S*_1_ regressors are largely independent of non-isotropic shape deformation and are therefore deemed reliable. The *S*_1_ effect has also been confirmed in a larger cohort of males (Gee et al., 2015).

#### 3.2.1 Biomechanical interpretation

**Fig. 12:**
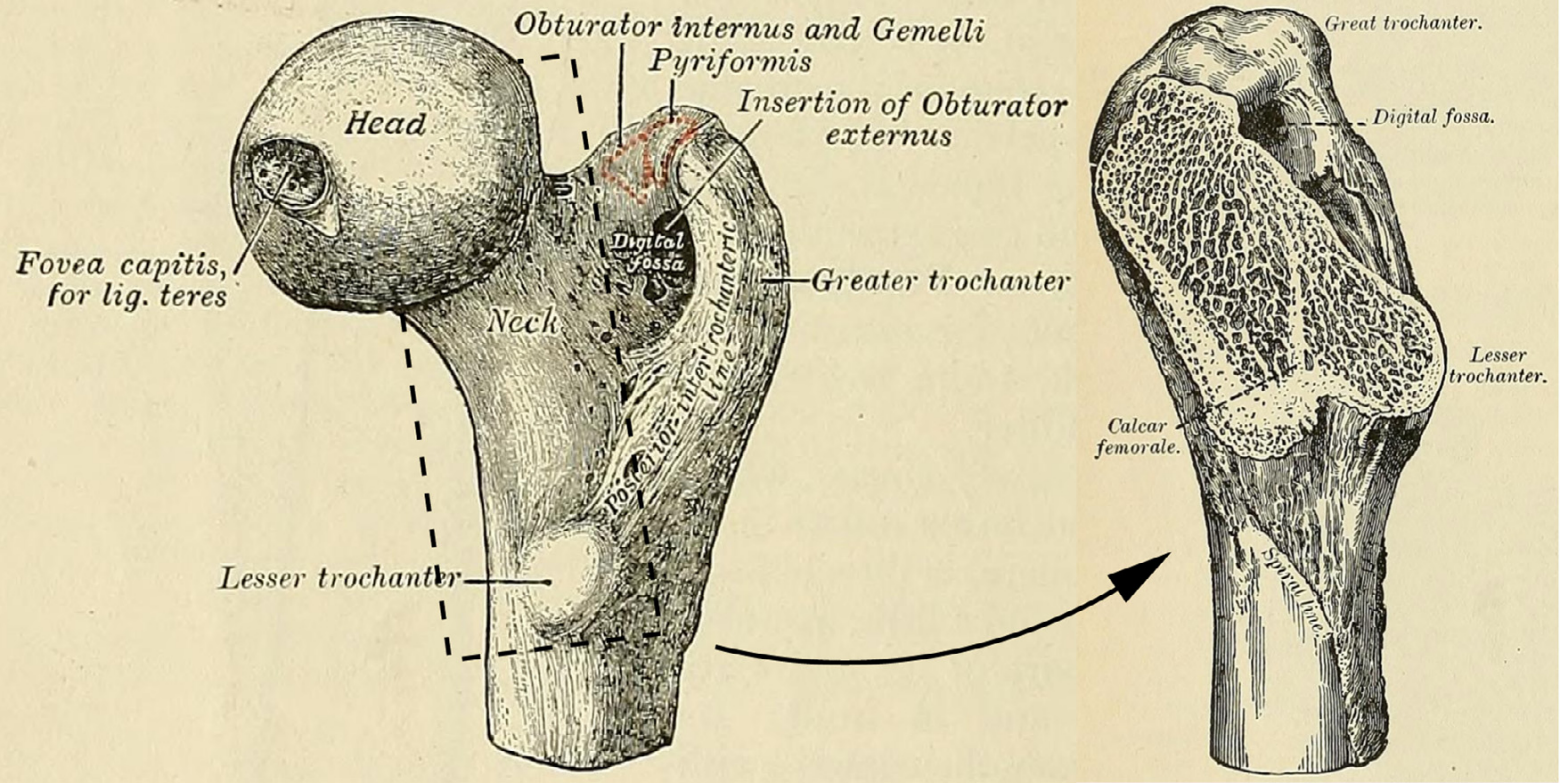
Anatomy of the proximal femur, with section exposing the calcar femorale. Adapted from Gray (1913), Figs. 176 and 182, retrieved 7 June 2017 from archive.org/details/anatomydescript00gray.

The *S*_2_ effect has not previously been described at this level of detail, and therefore warrants biomechanical interpretation. Of particular interest is the blue patch in Fig. 11(e), where CMSD is observed to increase in subjects with steeper femoral necks. As can be seen in Fig. 12, the dense, vertically orientated calcar femorale is situated at the convergence of multiple trabeculae whose function is to transmit load from the body, via the weight-bearing femoral head to the femoral shaft, the calcar acting as an internal compression buttress (Harty, 1957). This buttress, which has similar material properties to cortical bone (Li and Aspden, 1998), routes compressive load away from the lesser trochanter, which is left to transmit the large tensile and shear forces from the iliopsoas during hip flexion. The calcar joins the femoral cortex at precisely the blue patch in Fig. 11(e). That this patch is critical to transmission of force has been confirmed by attaching strain gauges to cadaveric femurs before and after surgical disruption of the calcar (Zhang et al., 2009). The blue patch can therefore be explained as a lifelong functional adaptation to variations in calcar loading that depend on the steepness of the femoral neck. Our results suggest increased calcar loading with steeper femoral necks, though we are unaware of any biomechanical simulations or measurements to support this hypothesis.

The red patches in Fig. 11(e) are more straightforward to explain.^4^ The bending moment across a steep (coxa valga) neck is less than across a small-angle (coxa vara) neck, so functional adaptation in the form of structural optimisation preferentially increases cortical bone in smaller angled necks. Voo et al. (2004) used finite element analysis to assess the effect of reducing the femoral neck angle, observing increased von Mises stress in regions aligned with the red patches in Fig. 11(e). Machado et al. (2014) used similar modelling to predict increased femoral neck bone mineral density in small-angle necks, while Ripamonti et al. (2014) observed precisely this phenomenon in a population of 315 males.

Similarly, the near global decrease in CMSD in stouter, less gracile femurs (the *S*_3_ effect in Fig. 11(f)) reflects bone’s intrinsic, evolutionary structural optimisation whereby as the neck diameter increases, the same section modulus can be maintained with less cortical bone (Bahari, Farahmand, Rouhi, Movahhedy, 2012, Rivadeneira, Zillikens, Laet, Hofman, Uitterlinden, Beck, Pols, 2007).

### 3.3 Homology-free registration

The proximal femur represents a challenging domain for surface registration. Apart from the general lack of distinguished features, especially anteriorly, there is also the arbitrary truncation at the shaft that makes registration in this area particularly difficult. We have argued that explicit landmarking is the only way to resolve these ambiguities and proceed with meaningful shape-texture analysis, but this begs the question as to whether homology-free registration might be feasible with less challenging surfaces. For example, when analysing lumbar vertebrae, a state-of-the-art, homology-free algorithm (e.g. Boyer et al. (2011)) would reliably align the various processes and pedicles, without the need for manual labelling. While this would most likely provide a reasonable basis for the statistical analysis of shape and texture, it would not enable the sort of sensitivity analysis we propose in Fig. 11(b). We therefore acknowledge a role for homology-free algorithms only inasmuch as they might *automatically* (and paradoxically) establish point and curve homologies, but we maintain that the homologies need to be explicit, not implicit, when analysing the dependence of surface texture on shape. This is not to discount the considerable value of homology-free methods in other contexts.

## 4 Conclusions

Statistical analysis of the relationship between surface texture and shape is sensitive to nuances of the surface registration algorithm, and therefore of questionable value unless correspondences are established explicitly using landmarks. We have presented a landmarking scheme for the human proximal femur, where all the landmarks can be identified in clinical CT data. Although there remains uncertainty in those regions of the surface some distance from the nearest landmark, we have suggested some heuristics for authenticating the statistical effects where possible. This holistic approach has revealed hitherto unreported dependencies between cortical mass and bone shape in the human proximal femur. We also observed how, when the regressor of interest is independent of shape, the analysis can be streamlined through the use of an homology-free registration algorithm, as long as said algorithm is purely shape-driven.

## Footnotes

1 www.mi.eng.cam.ac.uk/~rwp/stradwin.

2 www.mi.eng.cam.ac.uk/~ahg/wxRegSurf.

3 This allows us to study both size and shape simultaneously. In the formal terminology of morphometrics, we are therefore considering the *form* of the specimen, rather than just its *shape*.

4 These are the only patches in Fig. 11 that do not survive the inclusion of scanning site (Cambridge vs. Prague) in the GLM, though they do remain significant at the 7% level. This is because site happens to be weakly correlated with *S*_2_ (ρ=0.18), and SPM identifies only those effects that can be *uniquely* attributed to any particular regressor. Since a biomechanical explanation is far more plausible than any other cause, and entirely consistent with the work of Machado et al. (2014), Ripamonti et al. (2014) and Voo et al. (2004), we chose to omit site from the GLM in this instance. More generally, we acknowledge the limitations of inferential statistics in exploratory contexts such as this.
